# A nutritional assessment tool, GNRI, predicts sarcopenia and its components in type 2 diabetes mellitus: A Japanese cross-sectional study

**DOI:** 10.3389/fnut.2023.1087471

**Published:** 2023-02-01

**Authors:** Kaori Shiroma, Hayato Tanabe, Yoshinori Takiguchi, Mizuki Yamaguchi, Masahiro Sato, Haruka Saito, Kenichi Tanaka, Hiroaki Masuzaki, Junichiro J. Kazama, Michio Shimabukuro

**Affiliations:** ^1^Department of Diabetes, Endocrinology, and Metabolism, Fukushima Medical University School of Medicine, Fukushima, Japan; ^2^Department of Health and Nutrition, Faculty of Health and Nutrition, Okinawa University, Okinawa, Japan; ^3^Department of Nephrology and Hypertension, Fukushima Medical University School of Medicine, Fukushima, Japan; ^4^Division of Endocrinology, Diabetes, and Metabolism, Hematology, Rheumatology (Second Department of Internal Medicine), University of the Ryukyus, Okinawa, Japan

**Keywords:** aging, nutritional assessment, sarcopenia, type 2 diabetes, undernutrition

## Abstract

**Background:**

There are few reports evaluating the relationship between undernutrition and the risk of sarcopenia in type 2 diabetes mellitus (T2DM) patients.

**Objective:**

We investigated whether undernutritional status assessed by the geriatric nutritional risk index (GNRI) and controlling nutritional status (CONUT) were associated with the diagnosis of sarcopenia.

**Methods:**

This was a cross-sectional study of Japanese individuals with T2DM. Univariate or multivariate logistic regression analysis was performed to assess the association of albumin, GNRI, and CONUT with the diagnosis of sarcopenia. The optimal cut-off values were determined by the receiver operating characteristic (ROC) curve to diagnose sarcopenia.

**Results:**

In 479 individuals with T2DM, the median age was 71 years [IQR 62, 77], including 264 (55.1%) men. The median duration of diabetes was 17 [11, 23] years. The prevalence of sarcopenia was 41 (8.6%) in all, 21/264 (8.0%) in men, and 20/215 (9.3%) in women. AUCs were ordered from largest to smallest as follows: GNRI > albumin > CONUT. The cut-off values of GNRI were associated with a diagnosis of sarcopenia in multiple logistic regression analysis (odds ratio 9.91, 95% confidential interval 5.72–17.2), *P* < 0.001. The superiority of GNRI as compared to albumin and CONUT for detecting sarcopenia was also observed in the subclasses of men, women, body mass index (BMI) < 22, and BMI ≥ 22.

**Conclusions:**

Results showed that GNRI shows a superior diagnostic power in the diagnosis of sarcopenia. Additionally, its optimal cut-off points were useful overall or in the subclasses. Future large and prospective studies will be required to confirm the utility of the GNRI cut-off for undernutrition individuals at risk for sarcopenia.

## 1. Introduction

Sarcopenia is a progressive and generalized skeletal muscle disorder involving the accelerated loss of muscle mass and function (1–3). Type 2 diabetes mellitus (T2DM) is associated with an increased risk of sarcopenia (4–7), which can increase adverse outcomes, including functional decline, frailty, falls, and mortality (8, 9). Factors associated with sarcopenia in diabetes are age, HbA1c levels, visceral obesity, diabetic nephropathy, duration of diabetes, and chronic inflammation (5–7).

Malnutrition/undernutrition can be defined as “a state resulting from lack of intake or uptake of nutrition that leads to altered body composition (decreased fat-free mass) and body cell mass leading to diminished physical and mental function and impaired clinical outcome from disease” (10). In older adults with diabetes, irregular and unpredictable meal consumption can be linked to undernutrition (11). Also, therapeutic diets or the use of anti-diabetic agents may inadvertently lead to decreased food intake and contribute to unintentional weight loss and undernutrition (11). Malnutrition/undernutrition can increase the risk of sarcopenia in older adults with diabetes (12–14). Undernutrition is a nutritional disorder, whereas sarcopenia and frailty are nutrition-related conditions with complex and multiple pathogenic backgrounds (10). Therefore, undernutrition should be diagnosed, and optimal nutritional intervention combined with an exercise program needs to be considered to prevent sarcopenia (11–14). However, there are few reports evaluating the relationship between undernutrition and the risk of sarcopenia in individuals with diabetes (12–14). If we could predict sarcopenia by screening undernutrition, we can manage such individuals through an optimal nutritional and exercise program.

The Geriatric Nutritional Risk Index (GNRI) is a nutritional screening index which had been proposed to assess the nutrition-related risk originally for hospitalized elderly by Bouillanne et al. (15). The GNRI is a simple and objective index, allowing clinicians to assess patients readily based on height, weight and serum albumin level. GNRI is currently known as a prognostic predictor for patients with chronic diseases such as cardiovascular disease (16), chronic kidney diseases (17) or cancer (18). The Controlling Nutritional Status (CONUT) score, which is calculated based on the serum albumin level, total peripheral lymphocyte count and total cholesterol level, was developed as a screening tool for early detection of poor nutritional status (19). The GNRI and CONUT are often used in clinical practice because they are simpler than other nutritional indicators such as SGA (Subjective Global Assessment), MNA(Mini Nutritional Assessment), MUST (Malnutrition Universal Screening Tool), and NRS2002 (Nutritional Risk Screening) which require an interview of an expert (physicians, nurses, and/or dieticians) (20). Although there are previous reports between GNRI and sarcopenia in T2DM (13, 21), the clinical utility has not been clarified.

Therefore, we investigated whether undernutritional status as assessed by GNRI (15, 21, 22) and CONUT (19) are associated with the diagnosis of sarcopenia and its components. We also evaluated how the cut-off values of these screening tools could detect sarcopenia in Japanese individuals with T2DM.

## 2. Methods

### 2.1. Study design and subjects

This is a cross-sectional study in the part of the Fukushima Diabetes, Endocrinology, and Metabolism cohort (Fukushima DEM cohort). The DEM cohort recruited people with diabetes mellitus or high risk at diabetes who had visited the Department of Diabetes, Endocrinology, and Metabolism, Fukushima Medical University Hospital. The study protocol was approved by the Fukushima Medical University Ethics Committee (Number 29118). This study was conducted according to the Ethical Guidelines for Medical and Health Research Involving Human Subjects enacted by MHLW of Japan (https://www.mhlw.go.jp/file/06-Seisakujouhou-10600000Daijinkanboukouseikagakuka/0000069410.pdf and http://www.mhlw.go.jp/file/06-Seisakujouhou-10600000-Daijinkanboukouseikagakuka/0000080278.pdf) in line with the principles of the Declaration of Helsinki. The inclusion criteria of the current study were people with T2DM in the DEM cohort who had been recruited between January 2018 and December 2019. Among 795 patients who gave written informed consent, 240 patients who were either non-diabetic, had type 1 diabetes mellitus, or had secondary diabetes mellitus were excluded from the study (Supplementary Figure 1). After excluding missing data, 479 patients (265 men and 215 women) were included for a full analysis set. Various patient parameters, such as age, sex, history of diabetes, family and social history, medical checkup history, complications, medications, laboratory data, and all dates, were obtained from their paper and/or electrical medical records.

### 2.2. Data collection

Patients visited the hospital at 1–3 month intervals and continued receiving standardized treatment by endocrinologists/diabetologists. Trained staff measured the height, body weight, blood pressure, and waist circumference of participants. Questionnaires were provided to record the data on smoking (current or former smoker or not), drinking (former or every day, sometimes, rarely, or never), regular exercise (exercise to sweat lightly for over 30 min on each occasion, two times weekly), antihypertensive drug use, anti-hyperglycemic drug use, and lipid-lowering drug use. A participant was diagnosed with diabetes mellitus when the fasting plasma glucose level is ≥126 mg/dL, the HbA1c level is ≥6.5% (48 mmol/mol), or if the participant regularly uses anti-hyperglycemic drugs. A participant was diagnosed with hypertension if the systolic blood pressure was ≥140 mmHg, if the diastolic blood pressure was ≥90 mmHg, or if she/he regularly used antihypertensive drugs. A participant was diagnosed with dyslipidemia if the high-density lipoprotein (HDL) cholesterol level is <40 mg/dL (1.0 mmol/L), the low-density lipoprotein (LDL) cholesterol level is ≥140 mg/dL (3.6 mmol/L), the triglyceride level is ≥150 mg/dL (1.7 mmol/L), or if they regularly used lipid-lowering drugs. We calculated the estimated glomerular filtration rate (eGFR) using the Japanese formula (eGFR, mL/min/1.73 m^2^) = 194 × serum creatinine level (mg/dL) ^−1.094^ × age (years) ^−0.287^ (23).

Routine anthropometry and skeletal muscle mass, handgrip strength, walking speed, and body composition of the participants were assessed by trained staff, as previously reported (24). The waist circumference was measured at the level of the umbilicus (cm) in the standing position. Handgrip strength (kg) was measured using an isokinetic dynamometer (Smedley hand dynamometer) on both hands, and the values of the non-dominant arm were used. The fat and muscle composition in the whole body, trunk, arms, and legs were assessed using a body composition analyzer (InBody 770, InBody Japan Inc.) based on the segmental multifrequency bioelectrical impedance analysis (25, 26). The time required for walking 10 m was measured as described previously with slight modifications (27, 28). Fasting blood samples were collected after overnight fasting for ≥10 h and were assayed within 1 h using automatic clinical chemical analyzers. We excluded participants whose fasting blood samples could not be obtained. Nutritional intake indices were calculated using food frequency questionnaires as previously reported (24).

### 2.3. Nutritional assessment tool

#### 2.3.1. GNRI

The GNRI was calculated using the formula: GNRI = [14.89 × serum albumin level (g/dL)] + {41.7 × [current body weight (kg)/ideal body weight (kg)]} (15). In this study, the ideal body weight was determined from the participant's height and a BMI of 22 kg/m^2^. Following previous studies (22), the participants were separated into two groups by a cut-off of GNRI 98 which is a commonly used diagnostic level for undernutrition (GNRI < 98 or GNRI ≥ 98). Based on the calculation of a GNRI cut-off point for detecting sarcopenia, we also subdivided the participants into GNRI < 105 or GNRI ≥ 105 groups.

#### 2.3.2. CONUT

According to Ignacio de Ulíbarri J et al. (19), the CONUT score was obtained based on serum albumin concentration, cholesterol level, and lymphocyte count (Supplementary Table 1).

### 2.4. Assessment of sarcopenia

The definition and diagnosis of sarcopenia were based on the Asian Working Group for Sarcopenia (AWGS): 2019 Consensus Update on Sarcopenia Diagnosis and Treatment (2). In brief, “low muscle power” was defined as handgrip strength < 28 kg for men and < 18 kg for women; the criteria for “low physical performance” was walking speed < 1.0 m/s as evaluated by the time required for walking 10 m; and “low appendicular skeletal muscle mass (ASM)” was defined as a skeletal mass index (SMI) < 7.0 kg/m^2^ in men and < 5.7 kg/m^2^ in women. Sarcopenia is defined by low ASM and low muscle power or low physical performance.

## 3. Statistical analyses

Continuous and parametric values were expressed as mean ± standard deviation, and non-parametric values were expressed as median (first quartile–third quartile). Kolmogorov-Smirnov test was performed for normality. Group differences were analyzed by using two-tailed unpaired Student's *t*-test, one-way ANOVA or the Kruskal–Wallis test. Categorical values were expressed as percentages, and group differences were analyzed using the χ2 test.

We assessed the diagnostic value of serum albumin, BMI, GNRI, and CONUT by constructing the receiver operating characteristic (ROC) curve to distinguish between sarcopenia and non-sarcopenia in all participants, in men and women, in participants with BMI < 22 and BMI ≥ 22 and in diabetes duration < 5 and ≥ 5 years. The relevant area under the curve (AUC) was computed and compared as proposed by DeLong et al. (29). The optimal cut-off values were determined according to Youden's index, with the corresponding sensitivity, specificity, and accuracy at the cut-off value calculated and compared using the McNemar χ^2^ test.

Univariate or multivariate logistic regression analysis was performed to assess the association of GNRI with sarcopenia and its components indicated by the odds ratio (OR) with 95% confidential intervals (CI) in the unadjusted or adjusted models, respectively. The selection of covariates in the multivariate analysis was based on the items strongly associated with sarcopenia in previous studies (1–7). Model 1 was adjusted for age (year) and sex; model 2 was further adjusted for model 2 plus diabetes duration (year), HbA1c (%), and eGFR (ml/min/1.73 m^2^). Variables considered to have clinical implications were treated as potential variables to be controlled in model 3. BMI is a strong predictor of sarcopenia (1–7). Therefore, there was a risk of multicollinearity if BMI was included in the GNRI, which used current/ideal weight or current BMI/ BMI22 in the formula. When BMI and GNRI were simultaneously included in the multivariate logistic regression analysis to estimate sarcopenia, the VIF of BMI was 6.89 and the VIF of GNRI was 7.36, indicating a potential multicolineality with a VIF ≥ 5 (Supplementary Table 2). We therefore deleted BMI in the multivariate model using GNRI.

Statistical analyses were conducted using SPSS version 25 (SPSS, Inc., Chicago, Illinois, USA), EZR, or R (version 4.0.3). Values of *P* < 0.05 were considered statistically significant.

## 4. Results

### 4.1. General characteristics

#### 4.1.1. Men vs. women

The demographic and clinical characteristics of 479 types 2 diabetes (264 men and 215 women) are shown in Table 1. The median age was 71 years [62, 77], and 55.1% of the patients were men. The median duration of diabetes is 17 [11, 23] years. The prevalence of sarcopenia was 41/479 (8.6%) in all, 21/264 (8.0%) in men, and 20/215 (9.3%) in women. Men were older and had a longer duration of diabetes. Moreover, men had lower BMI and systolic blood pressure. In nutritional indices, men had a slightly lower GNRI but showed comparable values to women in the other undernutrition indices, such as GNRI < 98, GNRI < 105, CONUT, and CONUT ≥ 2. In the indices for sarcopenia, men had higher values in SMI, handgrip strength, and walking speed and showed lower frequencies of low handgrip strength and low walking speed. However, men showed comparable frequencies in low SMI and sarcopenia. Regarding comorbidities, the prevalence of coronary heart disease was higher in men, but hypertension, dyslipidemia, and stroke were comparable. The ratio of regular waking, smoking, and drinking was higher in men.

**Table 1:** General characteristics of men and women with type 2 diabetes mellitus.

	**All**	**Men**	**Women**	
**Parameters**	***n* = 479**	***n* = 264**	***n* = 215**	***P***
Age, years	71 [62, 77]	72 [64, 77]	69 [60, 76]	0.038
Men, *n* (%)	264 (55.1)	–	–	
Duration of diabetes, years	17 [11, 23]	18 [11, 24]	15 [9, 21]	0.008
**Anthropometry**				
Body weight, kg	65.1 [56.3, 77.9]	67.2 [59.1, 79.4]	61.2 [51.0, 74.5]	< 0.001
BMI, kg/m^2^	25.2 [22.2, 29.3]	24.6 [22.0, 28.1]	26.4 [22.8, 31.0]	0.002
Systolic blood pressure, mmHg	132 ± 18	131 ± 17	134 ± 18	0.028
Diastolic blood pressure, mmHg	73 ± 12	74 ± 12	73 ± 11	0.153
**Nutritional indices**				
GNRI, points	111 [105, 119]	110 [105, 117]	112 [106, 120]	0.009
Range	85–167	85–167	93–166	
GNRI < 98, *n* (%)	43 (9.0)	26 (9.8)	17 (7.9)	0.460
GNRI < 105, *n* (%)	111 (23.2)	65 (24.6)	46 (21.4)	0.405
CONUT, points	1.0 [0.0, 2.0]	1.0 [0.0, 2.0]	1.0 [0.0, 2.0]	0.124
Range	0–6	0–6	0–5	
CONUT ≥ 2, *n* (%)	210 (60.9)	105 (57.4)	105 (64.8)	0.158
**Indices for sarcopenia**				
Skeletal muscle index, kg/m^2^	7.1 [6.3, 7.9]	7.5 [7.0, 8.3]	6.4 [5.8, 7.1]	< 0.001
Handgrip strength, kg	30 [22.5, 39.0]	38 [31.5, 43.0]	23 [18.5, 27.0]	< 0.001
Walking speed, m/s	1.54 [1.33, 1.82]	1.67 [1.43, 1.82]	1.54 [1.25, 1.67]	< 0.001
Low SMI, *n* (%)	100 (20.9)	60 (22.7)	40 (18.6)	0.270
Low handgrip strength, *n* (%)	85 (17.7)	34 (12.9)	51 (23.7)	0.002
Low walking speed, *n* (%)	22 (4.6)	7 (2.7)	15 (7.0)	0.024
Sarcopenia, *n* (%)	41 (8.6)	21 (8.0)	20 (9.3)	0.600
**Comorbidities**				
Retinopathy, *n* (%)	130 (27.1)	77 (29.2)	53 (24.7)	0.269
eGFR < 60 ml/min/1.73 m^2^, *n* (%)	212 (44.3)	117 (44.3)	95 (44.2)	0.977
Hypertension, *n* (%)	403 (84.2)	223 (84.5)	180 (83.7)	0.823
Dyslipidemia, *n* (%)	409 (85.4)	218 (82.6)	191 (88.8)	0.054
Coronary heart disease, *n* (%)	74 (15.4)	56 (21.2)	18 (8.4)	< 0.001
Stroke, *n* (%)	40 (8.4)	26 (9.8)	14 (6.5)	0.189
**Life habits**				
Regular walking, *n* (%)	118 (24.8)	76 (29.0)	42 (19.6)	0.018
Current or ex-smoking, *n* (%)	254 (53.0)	202 (76.5)	52 (24.2)	< 0.001
Current or ex-drinking, *n* (%)	143 (29.9)	163 (61.7)	41 (19.1)	< 0.001
**Blood measurements**				
Albumin, g/dL	4.2 [4.0, 4.4]	4.3 [4.1, 4.5]	4.2 [4.0, 4.4]	0.083
AST, U/L	21 [17, 28]	22 [17, 29]	20 [17, 26]	0.014
ALT, U/L	19 [14, 29]	21 [14, 32]	17 [13, 26]	0.002
Fasting plasma glucose, mg/dL	131 [118, 154]	138 [122, 159]	127 [113, 145]	< 0.001
Glycated hemoglobin, %	6.9 [6.4, 7.4]	7.0 [6.5, 7.6]	6.8 [6.4, 7.3]	< 0.001
LDL-cholesterol, mg/dL	100 [82, 118]	97 [80, 115]	104 [85, 124]	< 0.001
HDL cholesterol, mg/dL	54 [46, 63]	51 [43, 60]	58 [49, 67]	< 0.001
Triglycerides, mg/dL	105 [73, 153]	108 [73, 162]	102 [72, 141]	0.247
Creatinine, mg/dl	0.83 [0.69, 1.00]	0.92 [0.79, 1.10]	0.70 [0.60, 0.84]	< 0.001
eGFR, ml/min/1.73 m^2^	63.2 [51.0, 76.0]	62.8 [51.0, 75.6]	64.1 [51.0, 76.2]	0.828
**Glucose-lowering drugs**				
Insulin, *n* (%)	132 (27.6)	76 (28.8)	56 (26.0)	0.504
GLP-1 receptor agonist, *n* (%)	34 (7.1)	26 (9.8)	8 (3.7)	< 0.001
Sulfonylurea, *n* (%)	49 (10.2)	34 (12.9)	15 (7.0)	0.034
Glinide, *n* (%)	118 (24.6)	76 (28.8)	42 (19.5)	0.019
Biguanide, *n* (%)	239 (49.9)	127 (48.8)	112 (52.1)	0.385
DPP4 inhibitor, *n* (%)	294 (61.4)	165 (62.5)	129 (60.0)	0.576
Pioglitazone, *n* (%)	147 (30.7)	82 (31.1)	65 (30.2)	0.845
α-glucosidase inhibitor, *n* (%)	90 (18.8)	63 (23.9)	27 (12.6)	0.002
SGLT2 inhibitor, *n* (%)	100 (20.9)	62 (23.5)	38 (17.7)	0.120
**Nutritional intake**				
Total energy intake (kcal/day)	2,010 [1,799–2,239]	2,212 [2,085–2,349]	1,795 [1,771–1825]	< 0.001
Total protein intake (g/day)	78.0 [70.3–85.2]	84.3 [80.4–88.6]	69.7 [67.7–72.6]	< 0.001
Total fat intake (g/day)	58.1 [54.7–63.6]	62.8 [59.8–66.5]	54.6 [53.8–55.8]	< 0.001
Total carbohydrate intake (g/day)	259 [244–295]	245 [238–249]	293 [269–312]	< 0.001
Protein energy (%)	15.4 [15.1–15.8]	15.3 [14.7–15.7]	15.6 [15.3–15.9]	< 0.001
Fat energy (%)	27.1 [25.6–27.5]	25.7 [24.7–26.8]	27.4 [27.2–27.7]	< 0.001

Data are expressed as median [25%, 75%], Mean ± SD, or number (%). GNRI, geriatric nutritional risk index; CONUT, controlling nutritional status; P, provability; BMI, body mass index; SMI, skeletal mass index; AST, aspartate aminotransferase; ALT, alanine aminotransferase; LDL, low-density lipoprotein; HDL, high-density lipoprotein; eGFR: estimated glomerular filtration rate; GLP-1, glucagon-like peptide-1; SGLT2, sodium-glucose cotransporter 2.

In blood measurements, albumin and eGFR were comparable between men and women. On the other hand, AST, ALT, glucose, HbA1c, and triglycerides were higher in men, and LDL- and HDL cholesterol were lower in men. Regarding anti-diabetic medication, the use of GLP-1 receptor agonist, sulfonylurea, glinide, and α-glucosidase inhibitor was higher in men, but the use of the other anti-diabetic medications was comparable.

#### 4.1.2. Sarcopenia– vs. sarcopenia+

**Figure 1:**
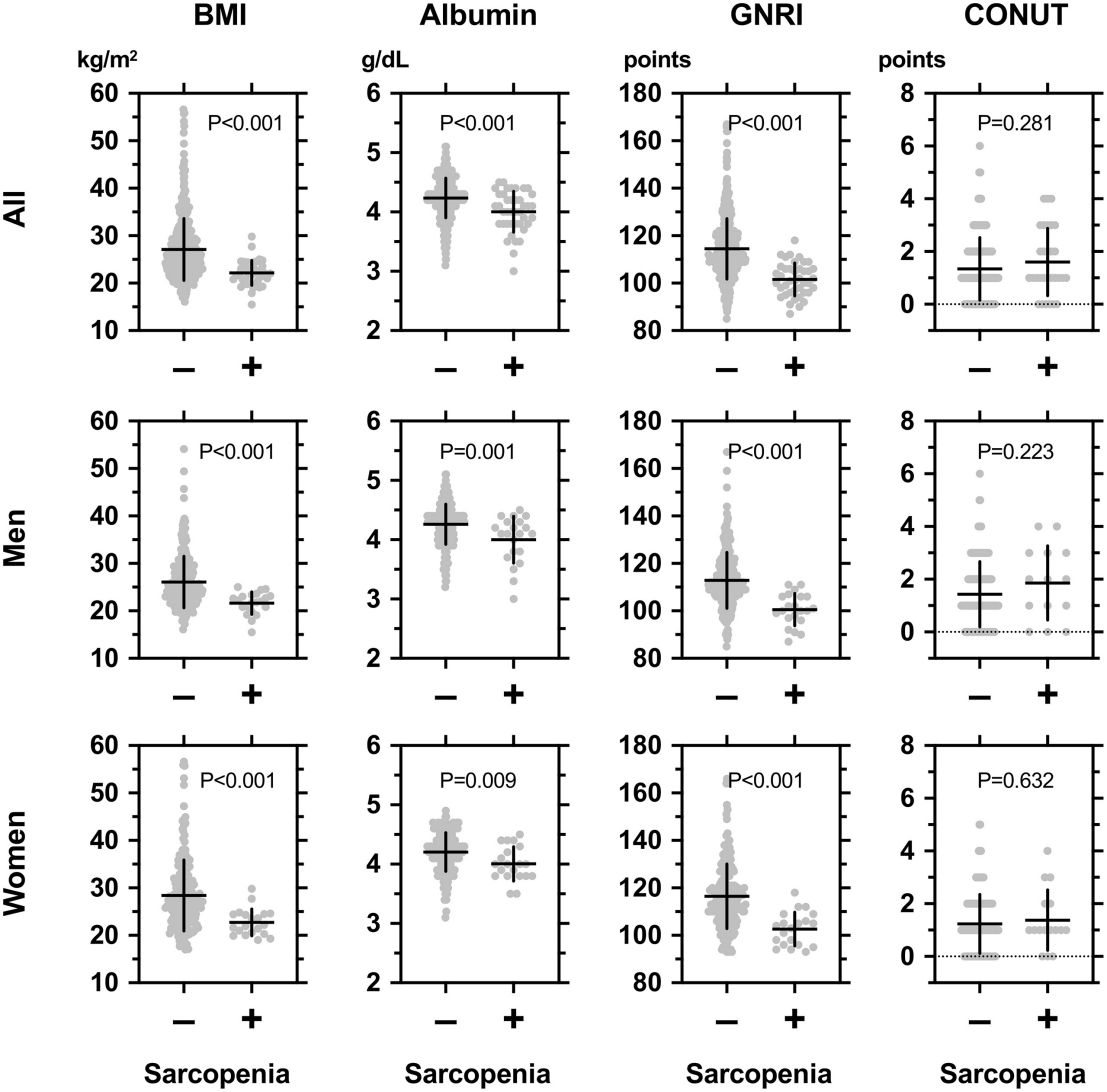
Comparisons among body mass index (BMI), serum albumin concentrations, geriatric nutritional risk index (GNRI), and controlling nutritional status (CONUT) between individuals with (+) or without (–) sarcopenia in all participants **(upper panel)**, men **(middle panel)**, and women **(lower panel)** with type 2 diabetes mellitus. The definition and diagnosis of sarcopenia were based on the Asian Working Group for Sarcopenia (AWGS): 2019 Consensus Update on Sarcopenia Diagnosis and Treatment (2). In brief, “low muscle power” was defined as handgrip strength < 28 kg for men and < 18 kg for women; the criterion for “low physical performance” was walking speed < 1.0 m/s as evaluated by the time required for walking 10 m; and “low appendicular skeletal muscle mass (ASM)” was defined as a skeletal mass index (BMI) < 7.0 kg/m^2^ in men and < 5.7 kg/m^2^ in women. Sarcopenia was defined by low skeletal mass index (SMI) and low muscle power or low physical performance.

The general characteristics of participants in the subgroups according to sarcopenia are shown in Table 2, left panel. The sarcopenia+ groups were older and had a longer duration of diabetes, lower BMI, and lower diastolic blood pressure. There was no difference in the ratios of men. As shown in Table 2 and Figure 1 (upper panel), the sarcopenia+ group showed a lower GNRI and higher frequencies in GNRI < 98 and GNRI < 105 while showing no difference in CONUT indices. The frequencies of comorbidities, except stroke, were comparable. Blood measurements and the use of glucose-lowering drugs were comparable, except that albumin and LDL-cholesterol were lower in the sarcopenia+ group. As shown in Figure 1 (middle panel), the sarcopenia+ groups in men and women showed lower values in BMI, albumin, and GNRI. Nutritional intake including total energy intake, total protein intake, total fat intake, total carbohydrate intake, protein energy, fat energy, and carbohydrate energy were not different between sarcopenia– vs. sarcopenia+.

**Table 2:** General characteristics of participants with type 2 diabetes mellitus in the subgroups accordingly to sarcopenia, GNRI, and CONUT.

	**Sarcopenia –**	**Sarcopenia +**		**GNRI < 105**	**GNRI ≥105**		**CONUT < 2**	**CONUT ≥2**	

**Parameters**	***n*** = **438**	***n*** = **41**	***P***	***n*** = **111**	***n*** = **368**	***P***	***n*** = **210**	***n*** = **135**	***P***
Age, years	70 [60, 75]	80 [74, 86]	< 0.001	73 [70, 79]	69 [59, 75]	< 0.001	68 [57, 74]	72 [63, 79]	0.003
Men, *n* (%)	243 (55.5)	21 (51.2)	0.600	65 (58.6)	199 (54.1)	0.405	105 (50.0)	78 (57.8)	0.158
Duration of diabetes, years	16 [11, 22]	21 [14, 28]	0.013	18 [11, 26]	16 [11, 22]	0.041	15 [9, 21]	18 [12, 24]	0.006
**Anthropometry**									
Body weight, kg	66.6 [58.1, 79.5]	51.8 [46.7, 56.7]	< 0.001	53.3 [47.7, 58.9]	69.4 [60.9, 81.9]	< 0.001	67.4 [57.6, 82.9]	63.6 [54.5, 77.1]	0.016
BMI, kg/m^2^	25.7 [22.8, 29.6]	22.0 [20.5, 23.9]	< 0.001	20.9 [19.8, 21.9]	27.1 [24.3, 30.8]	< 0.001	26.5 [23.3, 31.0]	25.3 [21.9, 28.3]	0.011
Systolic blood pressure, mmHg	132 ± 17	130 ± 19	0.550	131 [118, 142]	132 [121, 143]	0.133	133 ± 18	133 ± 16	0.764
Diastolic blood pressure, mmHg	74 ± 12	67 ± 10	< 0.001	71 [64, 79]	74 [67, 82]	0.005	74 ± 12	74 ± 11	0.673
**Nutritional indices**									
GNRI	112 [106, 120]	101 [96, 106]	< 0.001	100 [96, 102]	115 [110, 122]	< 0.001	114 [106, 122]	109 [102, 115]	< 0.001
Range	85–167	87–118		85–104	105–167		93–166	85–167	
GNRI < 98, *n* (%)	30 (6.8)	13 (31.7)	< 0.001	43 (38.7)	0 (0)	< 0.001	15 (7.1)	20 (14.8)	0.021
GNRI < 105, *n* (%)	85 (19.4)	26 (63.4)	< 0.001				43 (20.5)	38 (28.1)	0.101
CONUT, points	1.0 [0.0, 2.0]	1.0 [1.0, 3.0]	0.281	1.0 [0.5, 2.5]	1.0 [1.0, 2.0]	0.107	1.0 [0.0, 1.0]	2.0 [2.0, 3.0]	< 0.001
Range	0–6	0–4		0–6	0–5		0–1	2–6	
CONUT ≥ 2, *n* (%)	122 (38.7)	13 (43.3)	0.622	38 (46.9)	97 (36.7)	0.101			
**Indices for sarcopenia**									
Skeletal muscle index, kg/m^2^	7.2 [6.4, 8.0]	5.6 [5.3, 6.4]	< 0.001	6.3 [5.6, 6.9]	7.4 [6.5, 8.1]	< 0.001	7.2 [6.3, 8.1]	7.0 [6.2, 7.9]	0.277
Handgrip strength, kg	31.5 [24.0, 39.5]	17.5 [14.5, 22.3]	< 0.001	26.0 [19.0, 33.0]	31.5 [23.5, 40.4]	< 0.001	29.3 [22.0, 40.0]	29.0 [22.5, 37.5]	0.679
Walking speed, m/s	1.54 [1.43, 1.82]	1.25 [1.11, 1.43]	< 0.001	1.54 [1.33, 1.67]	1.54 [1.33, 1.82]	0.286	1.54 [1.33, 1.67]	1.54 [1.33, 1.67]	0.747
Low SMI, *n* (%)	59 (13.5)	41 (100)	< 0.001	24 (55.8)	76 (17.4)	< 0.001	36 (17.1)	33 (24.4)	0.098
Low handgrip, *n* (%)	44 (10.0)	41 (100)	< 0.001	33 (29.7)	52 (14.1)	< 0.001	35 (16.7)	31 (23.0)	0.147
Low walking speed, *n* (%)	16 (3.7)	6 (14.6)	0.001	5 (4.5)	17 (4.6)	0.960	10 (4.8)	7 (5.2)	0.859
Sarcopenia, *n* (%)	0 (0)	41 (100)		26 (23.4)	15 (4.1)	< 0.001	17 (8.1)	13 (9.6)	0.622
**Comorbidities**									
Retinopathy, *n* (%)	119 (27.2)	11 (26.8)	0.120	35 (31.5)	95 (25.9)	0.241	55 (26.2)	37 (27.4)	0.803
eGFR < 60 ml/min/1.73m^2^, *n* (%)	191 (43.6)	21 (51.2)	0.348	49 (44.1)	163 (44.4)	0.960	84 (40.0)	70 (51.9)	0.031
Hypertension, *n* (%)	370 (84.5)	33 (80.5)	0.504	80 (72.1)	323 (87.7)	< 0.001	177 (84.3)	117 (86.7)	0.543
Dyslipidemia, *n* (%)	374 (85.4)	35 (85.4)	0.997	78 (70.3)	331 (89.9)	< 0.001	182 (86.7)	113 (83.7)	0.445
Coronary heart disease, *n* (%)	68 (15.5)	6 (14.6)	0.880	11 (9.9)	63 (17.1)	0.065	25 (11.9)	23 (17.0)	0.179
Stroke, *n* (%)	33 (7.5)	7 (17.1)	0.035	8 (7.2)	32 (8.7)	0.619	17 (8.1)	11 (8.1)	0.986
**Life habits**									
Regular walking, *n* (%)	111 (25.5)	7 (17.5)	0.265	27 (24.8)	91 (24.8)	0.996	46 (22.1)	25 (18.7)	0.441
Current or ex-smoking, *n* (%)	235 (53.7)	19 (46.3)	0.370	19 (46.3)	235 (53.7)	0.370	114 (54.3)	67 (49.6)	0.398
Current or ex-drinking, *n* (%)	190 (43.4)	14 (34.1)	0.253	14 (34.1)	190 (43.4)	0.253	82 (39.0)	60 (44.4)	0.320
**Blood measurements**									
Albumin, g/dL	4.3 [4.1, 4.5]	4.0 [3.8, 4.3]	< 0.001	4.1 [3.8, 4.2]	4.3 [4.1, 4.5]	< 0.001	4.3 [4.1, 4.5]	4.2 [3.9, 4.4]	0.004
AST, U/L	21 [17, 28]	21 [18, 26]	0.779	21 [17, 27]	21 [17, 28]	0.683	20 [17, 28]	22 [18, 29]	0.057
ALT, U/L	19 [14, 29]	16 [13, 23]	0.064	16 [11, 23]	20 [14, 31]	< 0.001	19 [14, 30]	18 [12, 30]	0.172
Fasting plasma glucose, mg/dL	131 [118, 154]	130 [117, 150]	0.842	129 [114, 151]	132 [118, 154]	0.223	131 [117, 154]	132 [120, 155]	0.658
Glycated hemoglobin, %	6.9 [6.4, 7.5]	6.8 [6.3, 7.4]	0.429	6.8 [6.4, 7.4]	6.9 [6.4, 7.5]	0.747	6.9 [6.4, 7.6]	6.9 [6.4, 7.4]	0.172
LDL-cholesterol, mg/dL	102 [84, 119]	86 [78, 107]	0.024	97 [81, 114]	102 [83, 120]	0.105	110 [94, 129]	87 [75, 103]	< 0.001
HDL cholesterol, mg/dL	54 [46, 62]	51 [43, 71]	0.940	55 [44, 70]	53 [46, 62]	0.097	56 [46, 64]	50 [44, 62]	0.011
Triglycerides, mg/dL	104 [73, 154]	105 [70, 147]	0.531	82 [60, 129]	109 [78, 160]	< 0.001	117 [84, 179]	90 [66, 132]	< 0.001
Creatinine, mg/dl	0.84 [0.69, 1.01]	0.80 [0.68, 0.97]	0.409	0.83 [0.68, 1.03]	0.83 [0.69, 0.99]	0.898	0.82 [0.67, 1.00]	0.84 [0.71, 1.06]	0.117
eGFR, ml/min/1.73 m^2^	63.3 [51.1, 76.2]	59.9 [49.7, 73.6]	0.491	62.7 ± 19.7	63.3 ± 17.8	0.663	64.9 [50.9, 77.6]	59.5 [48.8, 74.9]	0.092
**Glucose-lowering drugs**									
Insulin, *n* (%)	121 (27.6)	11 (26.8)	0.913	43 (38.7)	89 (24.2)	0.003	53 (25.2)	45 (33.3)	0.104
GLP-1 receptor agonist, *n* (%)	34 (7.8)	0 (0)	0.064	4 (3.6)	30 (8.2)	0.102	15 (7.1)	9 (6.7)	0.865
Sulfonylurea, *n* (%)	43 (9.8)	6 (14.6)	0.330	12 (10.8)	37 (10.1)	0.818	25 (11.9)	11 (8.1)	0.265
Glinide, *n* (%)	106 (24.2)	12 (29.3)	0.471	33 (29.7)	85 (23.1)	0.155	46 (21.9)	31 (23.0)	0.818
Biguanide, *n* (%)	221 (50.5)	18 (43.9)	0.422	46 (41.4)	193 (52.4)	0.042	104 (49.5)	62 (45.9)	0.514
DPP4 inhibitor, *n* (%)	268 (61.2)	26 (63.4)	0.779	67 (60.4)	227 (61.7)	0.802	123 (58.6)	84 (62.2)	0.499
Pioglitazone, *n* (%)	137 (31.3)	10 (24.4)	0.360	17 (15.3)	130 (35.7)	< 0.001	64 (30.5)	43 (31.9)	0.787
α-glucosidase inhibitor, *n* (%)	79 (18.0)	11 (26.8)	0.168	29 (26.1)	61 (16.6)	0.024	40 (19.0)	17 (12.6)	0.115
SGLT2 inhibitor, *n* (%)	94 (21.5)	6 (14.6)	0.304	15 (13.5)	85 (23.1)	0.029	47 (22.4)	27 (20.0)	0.599
**Nutritional intake**									
Total energy intake (kcal/day)	2,012 [1,802–2,243]	1,926 [1,781–2,184]	0.157	2,039 [1,796–2,228]	1,997 [1,799–2,240]	0.941	1,951 [1,787–2,210]	2,031 [1,800–2,278]	0.187
Total protein intake (g/day)	78.2 [70.5–85.3]	76.9 [68.3–83.8]	0.323	78.7 [70.9–85.3]	77.7 [70.2–85.2]	0.549	77.0 [69.3–84.3]	79.0 [70.6–85.4]	0.133
Total fat intake (g/day)	58.2 [54.8–63.6]	57.3 [54.1–62.9]	0.404	59.3 [54.8–63.8]	58.0 [54.7–63.6]	0.639	57.3 [51.9–−54.8]	59.2 [55.3–63.7]	0.090
Total carbohydrate intake (g/day)	260 [245–296]	250 [241–292]	0.170	257 [243–297]	260 [245–295]	0.700	255 [242–293]	262 [246–302]	0.165
Protein energy (%)	15.4 [15.1–15.8]	15.5 [15.2–15.7]	0.756	15.5 [15.1–15.9]	15.4 [15.1–15.8]	0.220	15.4 [15.1–15.7]	15.3 [15.1–15.8]	0.843
Fat energy (%)	27.0 [25.6–27.5]	27.3 [25.9–27.6]	0.172	27.2 [25.7–27.6]	27.0 [25.6–27.5]	0.616	27.2 [25.6–27.5]	27.0 [25.5–27.5]	0.956
Carbohydrate energy (%)	53.7 [52.0–55.0]	53.5 [51.8–54.6]	0.359	53.6 [51.2–54.7]	53.7 [52.0–55.0]	0.234	53.8 [52.4–55.0]	53.7 [51.9–54.8]	0.492

Data are expressed as median [25%, 75%], Mean ± SD, or number (%). GNRI, geriatric nutritional risk index; CONUT, controlling nutritional status; P, provability; BMI, body mass index; SMI, skeletal mass index; AST, aspartate aminotransferase; ALT, alanine aminotransferase; LDL, low-density lipoprotein; HDL, high-density lipoprotein; eGFR: estimated glomerular filtration rate; GLP-1, glucagon-like peptide-1; SGLT2,sodium-glucose cotransporter 2.

#### 4.1.3. GNRI < 98 vs. GNRI ≥ 98

The group with GNRI < 98 was older and had a longer duration of diabetes, lower BMI, and lower systolic and diastolic blood pressure (Supplementary Table 3). There was no difference in terms of sex. They also showed a lower GNRI and a higher CONUT. Regarding sarcopenia, the group with GNRI < 98 had lower SMI, handgrip strength, higher frequencies of low SMI and low handgrip strength, and higher sarcopenia (30.2 vs. 6.4%, *P* < 0.001). They also showed lower values in albumin, ALT, LDL-cholesterol, and triglycerides. The use of α-glucosidase inhibitor was higher, and that of SGLT2 inhibitor was lower in this group.

#### 4.1.4. GNRI < 105 vs. GNRI ≥ 105

As described below, we found that GNRI < 105 was the cut-off for detecting sarcopenia in our participants. Therefore, we compared two subgroups accordingly (Table 2). The GNRI < 105 participants were older and had a longer duration of diabetes, lower BMI, and lower diastolic blood pressure but showed a comparable value in systolic blood pressure (Table 2). Regarding sarcopenia, the GNRI < 105 participants, as well as the GNRI < 98 participants, had lower values in SMI, handgrip strength, higher frequencies of low SMI and low handgrip strength, and higher sarcopenia. The use of insulin and α-glucosidase inhibitor was higher, and that of pioglitazone and SGLT2 inhibitor was lower in this group.

#### 4.1.5. CONUT < 2 vs. CONUT ≥ 2

The CONUT ≥ 2 group, which was estimated to be in an undernutrition state, was older and had a longer duration of diabetes and lower BMI but showed a comparable value in systolic and diastolic blood pressure as compared to the CONUT < 2 group (Table 2). However, the indices for sarcopenia were all comparable between the CONUT < 2 vs. CONUT ≥ 2 groups. The CONUT ≥ 2 group showed lower values in albumin, LDL- and HDL cholesterol, and triglycerides. The use of glucose-lowering drugs was similar between the two subgroups.

### 4.2. Diagnostic assessment of the nutritional indices for diagnosis of sarcopenia

**Figure 2:**
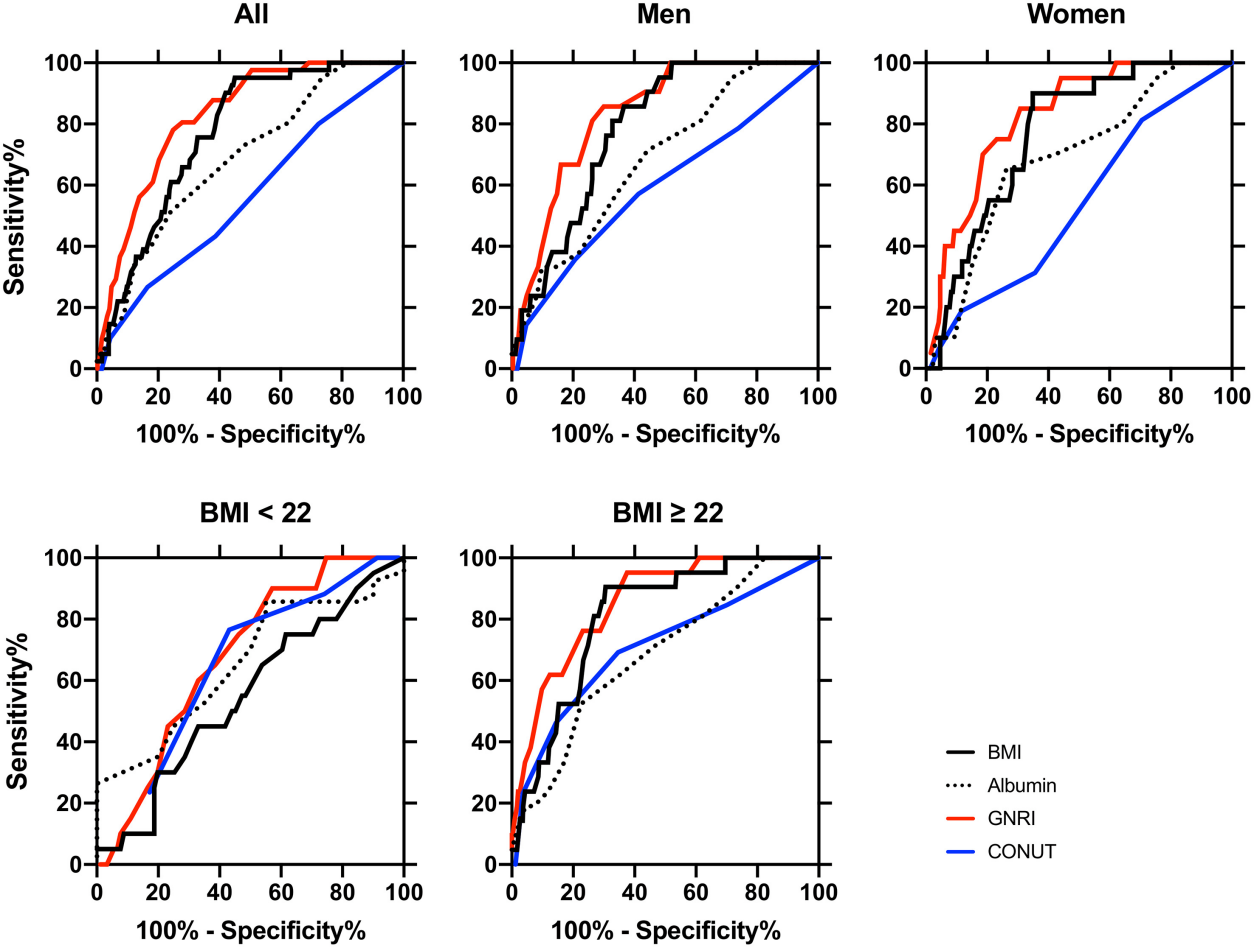
Receiver-operating characteristic (ROC) curves of the nutritional indexes for distinguishing between sarcopenia (*n* = 41) and non-sarcopenia (*n* = 438) patients in all participants (*n* = 479), in men (*n* = 264), in women (*n* = 215), BMI < 22 (*n* = 111), and BMI ≥ 22 (*n* = 368) subclasses. The area under the ROC curve (AUC) of body mass index (BMI, black lines), serum albumin (dotted lines), geriatric nutritional risk index (GNRI, red lines), and controlling nutritional status (CONUT, blue lines) were calculated for detecting diagnosis of sarcopenia, and statistical significance between AUC is shown in Table 3. The cut-off values, sensitivity, and specificity of these indices are also shown in Table 3. The definition and diagnosis of sarcopenia were based on the Asian Working Group for Sarcopenia (AWGS): 2019 Consensus Update on Sarcopenia Diagnosis and Treatment (2).

The AUCs and the optimal cut-off values of albumin, GNRI, and CONUT for detecting sarcopenia are shown in Figure 2 and Table 3. In all participants, the AUCs were ordered from largest to smallest as follows: GNRI > albumin > CONUT, showing that the diagnostic power of GNRI was superior to albumin. The AUC of GNRI was also statistically significant and was superior to albumin in all, men, women, BMI ≥ 22, and diabetes duration ≥ 5 years subgroups (Figure 2; Table 3).

**Table 3:** Diagnostic value of the nutritional indexes for distinguishing between sarcopenia and non-sarcopenia.

	**AUC**	**95% CI**	**Cut-off**	***P***	**P. vs. albumin**	***P* vs. GNRI**
**All**						
Albumin, g/dL	0.687	(0.607–0.766)	4.05	< 0.001	–	–
GNRI, points	0.827	(0.773–0.881)	105.8	< 0.001	< 0.001	–
CONUT, points	0.557	(0.448–0.667)	3.00	0.299	0.108	< 0.001
**Men**						
Albumin, g/dL	0.683	(0.573–0.792)	4.20	0.006	–	–
GNRI, points	0.833	(0.763–0.903)	106.9	< 0.001	< 0.001	–
CONUT, points	0.592	(0.425–0.725)	2.00	0.256	0.481	0.010
**Women**						
Albumin, g/dL	0.688	(0.574–0.803)	4.000	0.006	–	–
GNRI, points	0.827	(0.749–0.905)	105.8	< 0.001	0.012	–
CONUT, points	0.534	(0.390–0.677)	1.00	0.660	0.084	< 0.001
**BMI**<**22**						
Albumin, g/dL	0.655	(0.535–0.776)	3.80	0.030	–	–
GNRI, points	0.679	(0.568–0.790)	102.0	0.013	0.641	–
CONUT, points	0.654	(0.515–0.793)	1.00	0.055	0.673	0.729
**BMI** ≥**22**						
Albumin, g/dL	0.684	(0.572–0.797)	4.00	0.005	–	–
GNRI, points	0.852	(0.783–0.922)	112.1	< 0.001	< 0.001	–
CONUT, points	0.706	(0.544–0.869)	2.00	0.012	0.721	0.035
**Diabetes duration**<**5 years**						
Albumin, g/dL	0.321	(0.037–0.606)	4.40	0.316	–	–
GNRI, points	0.786	(0.593–0.979)	109.2	0.109	0.062	–
CONUT, points	0.435	(0.186–0.684)	1.00	0.828	0.102	0.015
**Diabetes duration** ≥**5 years**						
Albumin, g/dL	0.711	(0.632–0.789)	4.00	< 0.001	–	–
GNRI, points	0.831	(0.775–0.887)	105.8	< 0.001	< 0.001	–
CONUT, points	0.446	(0.332–0.559)	3.00	0.333	0.066	< 0.001

AUC, area under the curve; GNRI, geriatric nutritional risk index; CONUT, controlling nutritional status; CI, confidential intervals; P, provability.

### 4.3. Univariate and multivariate logistic regression analysis on the associations of nutritional indices with the diagnosis of sarcopenia and its components

Based on the cut-off values of the nutritional indices, we calculated the ORs for the diagnosis of sarcopenia and its components. In all participants (Table 4), univariate and multiple logistic regression analysis showed that albumin was associated with low handgrip strength and sarcopenia but not with low SMI and low walking speed. The cut-off values of GNRI of 98 and 105 were associated with the diagnosis of low SMI, low handgrip strength, and sarcopenia in univariate and multiple logistic regression analysis (models 1 and 2). The cut-off value of CONUT was not associated with the diagnosis of sarcopenia and its components.

**Table 4:** Univariate and multivariate logistic regression analysis on associations of cut-off of nutritional indices with a diagnosis of sarcopenia in the overall participants.

**Dependent variable**	**Unadjusted** ** OR (95% CI)**	***P***	**Model 1**	***P***	**Model 2**	***P***
**Independent variable: Albumin cut-off 4.05**						
Low SMI	1.43 (0.88–2.31)	0.147	1.06 (0.63–1.79)	0.829	1.06 (0.61–1.83)	0.832
Low handgrip strength	3.84 (2.35–6.26)	< 0.001	3.37 (1.93–5.89)	< 0.001	3.73 (2.08–6.67)	< 0.001
Low walking speed	2.45 (1.03–5.82)	0.042	1.64 (0.65–4.13)	0.294	2.09 (0.78–5.59)	0.141
Sarcopenia	3.33 (1.74–6.38)	< 0.001	2.39 (1.18–4.86)	0.016	2.65 (1.26–5.56)	0.010
**Independent variable: GNRI cut-off 98**						
Low SMI	5.99 (3.12–11.47)	< 0.001	4.26 (2.13–8.49)	< 0.001	4.09 (2.02–8.27)	< 0.001
Low handgrip strength	3.54 (1.82–6.87)	< 0.001	2.56 (1.22–5.37)	0.013	2.60 (1.22–5.53)	0.013
Low walking speed	1.65 (0.47–5.80)	0.438	1.08 (0.29–4.05)	0.911	1.35 (0.35–5.19)	0.664
Sarcopenia	6.31 (2.97–13.44)	< 0.001	4.64 (2.01–10.69)	< 0.001	4.67 (1.98–11.01)	< 0.001
**Independent variable: GNRI cut-off 105**						
Low SMI	11.74 (7.08–19.48)	< 0.001	5.77 (2.74–12.1)	< 0.001	0.75 (0.30–1.86)	< 0.001
Low handgrip strength	2.57 (1.56–4.25)	< 0.001	1.84 (1.05–3.23)	0.033	1.84 (1.04–3.25)	0.035
Low walking speed	0.97 (0.35–2.70)	0.960	0.62 (0.21–1.82)	0.388	0.66 (0.22–1.99)	0.457
Sarcopenia	7.19 (3.65–14.18)	< 0.001	5.77 (2.74–12.1)	< 0.001	9.91 (5.72–17.2)	< 0.001
**Independent variable: CONUT cut-off 3.0**						
Low SMI	1.60 (0.87–2.95)	0.131	1.50 (0.77–2.92)	0.240	1.59 (0.80–3.16)	0.190
Low handgrip strength	1.49 (0.78–2.86)	0.228	1.66 (0.79–3.50)	0.185	1.78 (0.83–3.79)	0.138
Low walking speed	1.59 (0.52–4.87)	0.416	1.78 (0.54–5.92)	0.348	1.70 (0.49–5.86)	0.400
Sarcopenia	1.80 (0.79–4.11)	0.163	1.76 (0.71–4.40)	0.226	1.81 (0.70–4.68)	0.222

Model 1: Age (year) and sex.

Model 1: Age (year) and sex Model 2: age (year), sex, diabetes duration (years), HbA1c (%), and eGFR (ml/min/1.73 m^2^).

OR, odds ratio; SMI, skeletal mass index; GNRI, geriatric nutritional risk index; CONUT, controlling nutritional status; CI, confidential intervals; P, provability.

Multiple logistic regression analysis (Model 2 in Table 4) on the associations of nutritional indices with the diagnosis of sarcopenia and its components in the subclasses of current participants is shown in Table 5 (ORs in unadjusted and Model 1 not shown). The cut-off values of albumin were associated with a diagnosis of sarcopenia only in women but not in the men, BMI < 22 and BMI ≥ 22 subclasses. The cut-off values of GNRI were associated with a diagnosis of sarcopenia in the men (105), women (105), BMI < 22 (102), BMI ≥ 22 (112), and diabetes duration ≥ 5 years (105) subgroups, but not in the diabetes duration < 5 years (Table 5). When using an originally reported low nutrition-related cut-off at 98 (15), the GNRI was associated with sarcopenia in the men, women, and diabetes duration ≥ 5 years subgroups but not in the BMI <22, BMI ≥ 22, and diabetes duration < 5 years subgroups. The cut-off values of CONUT were not associated with sarcopenia and its components in these subclasses, except in patients with BMI < 22.

**Table 5:** Multivariate logistic regression analysis (Model 2 in Table 4) on associations of cutoff of nutritional indices with diagnosis of sarcopenia.

	**Independent variable**							

	**Albumin**		**GNRI**		**GNRI**		**CONUT**	
**Dependent variable**	**Cutoff: 4.20**	***P***	**Cutoff: 98**	***P***	**Cutoff: 105**	***P***	**Cutoff: 2**	***P***
**Men**								
Low SMI	1.51 (0.79–2.90)	0.214	3.82 (1.52–9.57)	0.004	9.84 (4.73–20.47)	< 0.001	1.10 (0.55–2.19)	0.796
Low hand grip strength	1.86 (0.81–4.29)	0.143	2.69 (0.99–7.31)	0.053	2.48 (1.10–5.59)	0.028	1.18 (0.51–2.75)	0.699
Low walking speed	0.36 (0.07–1.97)	0.236	1.54 (0.23–10.19)	0.656	0.98 (1.18–5.23)	0.983	0.94 (0.17–5.16)	0.940
Sarcopenia	2.15 (0.76–6.13)	0.151	3.76 (1.23–11.55)	0.021	5.29 (1.90–14.68)	< 0.001	0.90 (0.32–2.56)	0.843
	**Independent variable**							
	**Albumin**		**GNRI**		**GNRI**		**CONUT**	
**Dependent variable**	**Cutoff: 4.00**	***P***	**Cutoff: 98**	***P***	**Cutoff: 105**	***P***	**Cutoff: 1**	***P***
**Women**								
Low SMI	2.15 (0.93–4.95)	0.073	5.29 (1.67–16.78)	0.005	12.04 (4.98–29.14)	< 0.001	1.30 (0.52–3.26)	0.575
Low hand grip strength	5.42 (2.30–12.77)	< 0.001	2.35 (0.73–7.53)	0.152	1.44 (0.64–3.24)	0.379	1.22 (0.52–2.87)	0.645
Low walking speed	2.99 (0.84–10.67)	0.093	0.84 (0.09–7.74)	0.877	0.44 (0.09–2.23)	0.321	0.71 (0.19–2.64)	0.608
Sarcopenia	7.69 (2.21–26.67)	< 0.001	7.07 (1.75–28.58)	0.006	7.71 (2.24–26.54)	< 0.001	0.58 (0.16–2.09)	0.407
	**Independent variable**							
	**Albumin**		**GNRI**		**GNRI**		**CONUT**	
**Dependent variable**	**Cutoff: 3.70**	***P***	**Cutoff: 98**	***P***	**Cutoff: 102**	***P***	**Cutoff: 1**	***P***
**BMI**<**22**								
Low SMI	0.22 (0.06–0.77)	0.018	0.95 (0.41–2.21)	0.907	1.11 (0.50-2.46)	0.802	0.97 (0.37–2.56)	0.958
Low hand grip strength	2.22 (0.59–8.35)	0.240	3.51 (1.25–9.86)	0.017	5.32 (1.61–17.56)	0.006	0.55 (0.17–1.83)	0.333
Low walking speed	9.42 (0.57–154.68)	0.116	5.96 (0.46–76.65)	0.171	–		0.24 (0.01–7.28)	0.415
Sarcopenia	0.86 (0.18–4.10)	0.848	3.01 (0.10–9.08)	0.051	7.73 (1.77–33.78)	0.007	0.23 (0.05–0.97)	0.045
Low SMI	1.18 (0.51–2.72)	0.736	3.49 (0.29–42.05)	0.326	11.69 (3.34–40.50)	< 0.001	0.74 (0.32–1.73)	0.485
Low hand grip strength	3.44 (1.68–7.08)	< 0.001	6.59 (0.62–70.29)	0.119	1.53 (0.74–3.16)	0.250	1.60 (0.79–3.24)	0.189
Low walking speed	1.80 (0.57–5.65)	0.317	–		0.35 (0.11–1.13)	0.079	1.14 (0.38–3.44)	0.817
Sarcopenia	2.56 (0.89–7.36)	0.081	12.34 (0.72–212.73)	0.084	7.15 (1.45–35.33)	0.016	1.09 (0.37–3.22)	0.873
	**Independent variable**							
	**Albumin**		**GNRI**		**GNRI**		**CONUT**	
**Dependent variable**	**Cutoff: 4.4**	***P***	**Cutoff: 98**	***P***	**Cutoff: 105**	***P***	**Cutoff: 1**	***P***
**Diabetes duration**<**5 years**								
Low SMI	0.08 (0.00–1.63)	0.100	–		14.10 (0.93–214.6)	0.057	34.1 (0.17–7,032.3)	0.195
Low hand grip strength	0.19 (0.01–3.61)	0.269	12.46 (0.27–582.9)	0.199	–		–	
Low walking speed	–		–		–		–	
Sarcopenia	0.02 (0.00–2.65)	0.113	–		67.23 (0.26–17,568.6)	0.138	–	
	**Independent variable**							
	**Albumin**		**GNRI**		**GNRI**		**CONUT**	
**Dependent variable**	**Cutoff: 4.00**	***P***	**Cutoff: 98**	***P***	**Cutoff: 105**	***P***	**Cutoff: 3**	***P***
**Diabetes duration** ≥**5 years**								
Low SMI	0.90 (0.48–1.70)	0.752	4.73 (2.27–9.83)	< 0.001	9.39 (5.31–16.61)	< 0.001	1.67 (0.80–3.46)	0.171
Low hand grip strength	2.89 (1.55–5.39)	< 0.001	2.31 (1.07–4.96)	0.032	1.58 (0.87–2.85)	0.131	1.46 (0.66–3.23)	0.350
Low walking speed	1.97 (0.72–5.41)	0.186	1.39 (0.36–5.40)	0.635	0.68 (0.22–2.10)	0.506	1.39 (0.39–4.91)	0.612
Sarcopenia	2.71 (1.23–5.98)	0.014	5.20 (2.15–12.60)	< 0.001	5.55 (2.51–12.30)	< 0.001	1.76 (0.63–4.94)	0.280

Data are odds ratio (95% confidential intervals) as Model 2 in Table 4 corrected for age (years), sex, diabetes duration (years), HbA1c (%), and eGFR (ml/min/1.73 m^2^).

GNRI, geriatric nutritional risk index; CONUT, controlling nutritional status; P, provability; SMI, skeletal mass index; BMI, skeletal mass index.

## 5. Discussion

The current study investigated whether undernutrition status, as assessed by GNRI, CONUT, and albumin, is associated with the diagnosis of sarcopenia and its components. We also determined the diagnostic power of the cut-off values for detecting sarcopenia in Japanese individuals with T2DM. We obtained two major findings. First, the cut-off values of albumin and GNRI 98 and 105, but not that of CONUT, were associated with a diagnosis of sarcopenia in the overall, men and women groups (Tables 4, 5). The AUC of GNRI was significantly larger than those of albumin and CONUT, indicating that the diagnostic power of GNRI was superior to both (Table 3). Second, the superiority of GNRI as compared to albumin and CONUT for sarcopenia was also observed in the subclasses. The AUCs of GNRI was significantly larger than that of albumin and CONUT in these subclasses (Table 3). The cut-off values of GNRI were associated with a diagnosis of sarcopenia in the BMI < 22 (102), BMI ≥ 22 (112), and diabetes duration ≥ 5 years (105) subgroups (Table 5). The GNRI cut-off of 98, which was commonly used as the diagnostic level for undernutrition (29), was not associated with sarcopenia in the BMI < 22 and BMI ≥ 22 subgroups. The cut-off values of albumin and CONUT were not associated with a diagnosis of sarcopenia.

To our knowledge, this study first provides us with a comparison of the diagnostic utility of the indexes commonly used in the nutritional assessment of people with T2DM. This study also determined the cut-off values of the nutritional indexes in sarcopenia and made a comparison to show their superiority or inferiority. We found that GNRI, a simple screening formula for undernutrition, shows a superior diagnostic power. Future large and prospective studies will be required to confirm the utility of the GNRI cut-off for undernutrition individuals at risk for sarcopenia.

### 5.1. GNRI and diagnosis of sarcopenia and its components

There are reports on the associations between the nutritional indicators such as SGA, MNA, MUST, and NRS2002 and diagnosis of sarcopenia (20, 30, 31). These reports repeatedly indicated that malnutrition determined by these indices and diagnosis of sarcopenia are closely linked (20, 30, 31). However, these indicators require interviews for history of body weight and dietary assessment, limiting clinical application. There are more simpler indices such as albumin and prealbumin. Xiu et al. reported that low prealbumin levels were associated with an increased risk for sarcopenia in older men with T2DM (32). However, prediction of undernutrition using these simple indices may be limited to some extent by potential confounding factors such as other clinical conditions (20, 30, 31). In our study, we adopted GNRI (15, 21, 22), CONUT (19), and albumin which are easily available in daily clinical practice for the assessment of undernutrition.

The cut-off values of GNRI were associated with a diagnosis of sarcopenia in multiple logistic regression analysis after correcting for potential cofounders. There are previous reports on the association between GNRI with the diagnosis of sarcopenia and its components. In Korean patients on hemodialysis, a GNRI of 97–101 (OR 0.064, 95% CI 0.005–0.883, compared to GNRI ≤ 96, *p* = 0.040) was associated with a lower sarcopenia risk (33). Xiang et al. reported that the overall diagnostic performance was the best for mid-arm circumference, followed by GNRI, calf circumference, BMI, and the worst for triceps skinfold thickness and albumin in detecting sarcopenia in community-dwelling Chinese adults aged 50 or older (34). The following two reports agreed with our findings, showing the association between low GNRI and the diagnosis of sarcopenia (13, 21). Takahashi et al. reported that a GNRI < 98 was related to the prevalence of sarcopenia [adjusted odds ratio, 4.88 (95%CI: 1.88–12.7), *p* = 0.001] in Japanese patients with T2DM (13). Matsuura et al. reported that a higher GNRI was associated with a lower risk of sarcopenia in older men and women with diabetes [multivariate-adjusted OR, 0.892; 95% CI, 0.839–0.948 for male; adjusted OR, 0.928; 0.876–0.982 for female] (21). However, the GNRI threshold and its relevance in subclasses for sarcopenia were not considered in the two studies. We further assessed the diagnostic utility of the cut-off values of GNRI to detect sarcopenia. The GNRI cut-off values of 105 and 98 were associated with a diagnosis of sarcopenia similarly in all participants and in the men and women subclasses. However, a GNRI of 102 in patients with BMI < 22 and a GNRI of 112 in patients with BMI ≥ 22, but not that of 98, were associated with a diagnosis of sarcopenia. Collectively, it is suggested that the optimal cut-off values of GNRI depend on the clinical characteristics of the target population.

### 5.2. Potential mechanisms by which GNRI predicts sarcopenia

There were reports indicating the association between low GNRI and low muscle power, and low muscle mass (22, 35). In Chinese elderly people, a low GNRI was associated with a higher incidence of low muscle mass (34). In Italian institutionalized elderly, GNRI was correlated with arm muscle area, handgrip strength, and handgrip strength/arm muscle area (22). Compared to other indices such as the ESPEN, GLIM, or SGA criteria, GNRI appears simple but still considers the serum albumin level in addition to current and ideal body weight (15).

The mechanisms by which low GNRI correlates with sarcopenia may include the lack of supply of muscle building blocks due to undernutrition and the involvement of chronic inflammation of muscle due to undernutrition (14, 36, 37). In Germany's older patients, a higher risk GNRI was associated with increased CRP levels (*p* < 0.05) and low lymphocyte counts (*p* < 0.05) after multivariable adjustment (36). Subclinical catabolic and inflammatory states, which are associated with chronic disease, led to increased production of catabolic cytokines, increased muscle catabolism, and decreased appetite with a negative effect on albumin levels (38–40). A reduction in serum albumin can therefore be a consequence of poor nutritional status or inflammation/disease (38–40).

Although low albumin has long been recognized as a crude indicator of undernutrition status (41), it is an unreliable indicator of nutritional status because it may be more related to inflammation or hydration status than to malnutrition (15, 42, 43). The GNRI was developed by Bouillanne et al. in 2005 to provide a prognostic nutritional index that enables quantitative determination of the risk of nutrition-related morbidity and mortality in elderly patients at admission into a geriatric hospital (15). They described that GNRI is not an index of malnutrition, but it is a “nutrition-related” risk index because GNRI scores are correlated to a severity score that considers nutritional status-related complications such as bedsores and infections (15). Importantly, GNRI is also based on measurements of weight loss, which are strong independent risk factors for comorbidities and mortality in older persons (44, 45). Applying the status of weight in the formula, GNRI can be a better predictor than serum albumin for low SMI and sarcopenia in the elderly with T2DM with a median age of 80 [IQR 74, 86]. The CONUT formula includes blood biomarkers such as serum albumin concentration, cholesterol level, and lymphocyte count but does not include body composition measures such as BMI (19). Because assessment of muscle mass is critical in considering the diagnosis of sarcopenia, the diagnostic power of CONUT can be low for detecting sarcopenia. The cut-off values of GNRI were rather different in the subclasses of T2DM participants. The GNRI cut-off values were associated with low SMI and diagnosis of sarcopenia in men and women (105), and BMI ≥ 22 (112) subclasses. While the GNRI cut-off value of 102 was associated with low handgrip strength and diagnosis of sarcopenia in patients with BMI < 22. When using an originally reported low nutrition-related cut-off of 98 (15), GNRI was associated with sarcopenia in men and women subclasses but not in the BMI < 22 and BMI ≥ 22 subclasses. The cut-off values of CONUT were not associated with a diagnosis of sarcopenia and its components in these subclasses, except in the diagnosis of sarcopenia in BMI < 22. The individual components of the CONUT score are shown in Supplementary Table 1. The distribution of scoring of lymphocyte count, total cholesterol, and albumin was not different between the sarcopenia– and sarcopenia+ groups. As discussed above, if the status of weight in the formula is applied, GNRI could be a better predictor for sarcopenia in the subclasses BMI < 22 and BMI ≥ 22.

### 5.3. Limitations

Our study had some limitations. First, because this study was conducted at a single university hospital, there may be a bias toward patients with a high risk of developing sarcopenia. Second, the number of patients and duration of observation might have been insufficient to assess the development of sarcopenia. Third, this was a cross-sectional observational study, and low albumin followed by lower GNRI and the prevalence of low SMI and sarcopenia are mutually well-correlated. Therefore, we could not determine a cause-and-result relationship. Further large-scale longitudinal studies are needed to corroborate the results of this study.

## 6. Conclusion

Results indicated that GNRI shows a superior diagnostic power in the diagnosis of sarcopenia. Additionally, its optimal cut-off points were useful as compared to a GNRI cut-off of 98, which is a commonly used diagnostic level for undernutrition. Future large and prospective studies will be required to confirm the utility of the cut-off for undernutrition individuals at risk for sarcopenia.

## Data availability statement

The raw data supporting the conclusions of this article will be made available by the authors, without undue reservation.

## Ethics statement

The studies involving human participants were reviewed and approved by the Fukushima Medical University Ethics Committee. The patients/participants provided their written informed consent to participate in this study.

## Author contributions

KS and MSh contributed to the concept and design of the study and analyzed the data. HT, YT, MY, MSa, HS, and MSh participated in data collection. KS and MSh wrote the first draft with input from HT, YT, MY, MSa, HS, KT, HM, and JJK. All authors contributed to the discussion and approved the final manuscript.
