# Supplementary material for: A nutritional assessment tool, GNRI, predicts sarcopenia and its components in type 2 diabetes mellitus: A Japanese cross-sectional study
Source: Front Nutr. 2023 Feb 1;10:1087471. doi: 10.3389/fnut.2023.1087471 (PMC9928854; doi:10.3389/fnut.2023.1087471)
Supplement: Supplementary file 1 [file Table_1.pdf]

Supplemental Table 1. Individual components of the CONUT score

Lymphocyte (/µL)	Ly ≥1600	1200≤ Ly <1600	800≤ Ly <1200	Ly <800	
Scoring	0	1	2	3	P
Overall	51.6%	29.3%	16.2%	2.9%	
Sarcopenia –	52.1%	28.6%	16.2%	3.2%	0.632
Sarcopenia +	46.7%	36.7%	16.7%	0.0%	
Men	53.6%	26.2%	17.5%	2.7%	0.585
Women	49.4%	32.7%	14.8%	3.1%	
BMI < 22	38.7%	32.0%	22.7%	6.7%	0.015
BMI ≥ 22	55.2%	28.5%	14.4%	1.9%	

Total cholesterol(mg/dL)	TC ≥180	140≤ TC <180	100≤ TC <140	TC <100	
Scoring	0	1	2	3	P
Overall	48.7%	42.6%	8.7%	0.0%	
Sarcopenia –	49.8%	41.9%	8.3%	0.0%	0.331
Sarcopenia +	36.7%	50.0%	13.3%	0.0%	
Men	43.7%	43.2%	13.1%	0.0%	0.005
Women	54.3%	42.0%	3.7%	0.0%	
BMI < 22	48.0%	45.3%	6.7%	0.0%	0.730
BMI ≥ 22	48.9%	41.9%	9.3%	0.0%	

Albumin (g/dL)	Alb ≥3.5	3.0≤ Alb <3.5	2.5≤ Alb <3.0	Alb <2.5	
Scoring	0	2	4	6	P
Overall	97.1%	2.9%	0.0%	0.0%	
Sarcopenia –	97.5%	2.5%	0.0%	0.0%	0.213
Sarcopenia +	93.3%	6.7%	0.0%	0.0%	
Men	96.2%	3.8%	0.0%	0.0%	0.346
Women	98.1%	1.9%	0.0%	0.0%	
BMI < 22	96.0%	4.0%	0.0%	0.0%	0.458
BMI ≥ 22	97.4%	2.6%	0.0%	0.0%	

Ly = lymphocyte count, TC = total cholesterol, Alb = albumin.
