# Supplementary material for: A nutritional assessment tool, GNRI, predicts sarcopenia and its components in type 2 diabetes mellitus: A Japanese cross-sectional study
Source: Front Nutr. 2023 Feb 1;10:1087471. doi: 10.3389/fnut.2023.1087471 (PMC9928854; doi:10.3389/fnut.2023.1087471)
Supplement: Supplementary file 2 [file Table_2.pdf]

Supplemental Table 2. Multivariate logistic regression analysis on associations of GNRI cutoff for diagnosis of sarcopenia in the overall participants

Dependent variable: Sarcopenia

Independent variable	Standardized	P	VIF
Age (year)	0.219	<0.001	1.815
Sex	-0.052	0.243	1.816
Diabetes duration (year)	-0.002	0.972	1.346
HbAic (%)	0.013	0.978	1.106
eGFR (ml/min/1.73m^2)	0.001	0.105	1.205
GNRI, point	-0.408	<0.001	7.356
BMI (kg/㎡)	0.005	0.029	6.888

VIF: variance inflation factor

Independent variable: GNRI cut-off 98

Dependent variable	Model 2 in Table 4	P	Model 3	P
Low SMI	4.09 (2.02 - 8.27)	<0.001	0.53 (0.23 - 1.23)	0.141
Low handgrip strength	2.60 (1.22 - 5.53)	0.013	3.39 (1.49 - 7.69)	0.004
Low walking speed	1.35 (0.35 - 5.19)	0.664	3.24 (0.73 - 14.40)	0.123
Sarcopenia	4.67 (1.98 - 11.01)	<0.001	1.95 (0.74 - 5.13)	0.175

Independent variable: GNRI cut-off 105

Dependent variable	Model 2 in Table 4	P	Model 3	P
Low SMI	0.75 (0.30 - 1.86)	<0.001	1.73 (0.84 - 3.54)	0.140
Low handgrip strength	1.84 (1.04 - 3.25)	0.035	2.83 (1.14 - 5.66)	0.003
Low walking speed	0.66 (0.22 - 1.99)	0.457	1.46 (0.41 - 5.18)	0.555
Sarcopenia	9.91 (5.72 - 17.2)	<0.001	3.13 (1.19 - 8.25)	0.021

Model 2: age (year), sex, diabetes duration (years), HbA1c (%), and eGFR (ml/min/1.73m^2)

Model 3: age (year), sex, diabetes duration (years), HbA1c (%), eGFR (ml/min/1.73m^2), and BMI (kg/㎡)
