# Supplementary material for: A nutritional assessment tool, GNRI, predicts sarcopenia and its components in type 2 diabetes mellitus: A Japanese cross-sectional study
Source: Front Nutr. 2023 Feb 1;10:1087471. doi: 10.3389/fnut.2023.1087471 (PMC9928854; doi:10.3389/fnut.2023.1087471)
Supplement: Supplementary file 3 [file Table_3.pdf]

Supplemental Table 3. General characteristics of participants with type 2 diabetes mellitus in the subgroups accordingly to GNRI

Parameters	GNRI <98 n=43	GNRI ≥98 n=436	
Age, years	76 [70, 80]	71 [60, 75]	<0.001
Men, n (%)	26 (60.5)	238 (54.6)	0.464
Duration of diabetes, years	20 [13, 34]	16 [11, 22]	0.005
**Anthropometry**			
Body weight, kg	52.1 [45.3, 58.0]	66.7 [58.1, 79.5]	<0.001
BMI, kg/m^2	20.3 [18.9, 21.5]	25.8 [23.2, 29.7]	<0.001
Systolic blood pressure, mmHg	125 ± 21	133 ± 17	0.006
Diastolic blood pressure, mmHg	67 ± 12	74 ± 11	<0.001
**Nutritinal indices**			
GNRI	94[91, 96]	112 [107, 120]	<0.001
Range	85-97	98-167	
CONUT, points	2.0 [1.0, 3.0]	1.0 [0.0, 2.0]	0.002
Range	0-6	0-5	
CONUT ≥ 2, n (%)	20 (57.1)	115 (37.1)	0.021
**Indices for sarcopenia**			
Skeletal muscle index, kg/m^2	6.3[5.4, 7.1]	7.2 [6.4, 8.0]	<0.001
Hand grip strength, kg	24.5 [17.5, 32.5]	30.5 [23.0, 39.5]	<0.001
Walking speed, m/sec	1.43 [1.18, 1.67]	1.54 [1.36, 1.82]	0.060
Low SMI, n (%)	24 (55.8)	76 (17.4)	<0.001
Low hand grip strength, n (%)	17 (39.5)	68 (15.6)	<0.001
Low walking speed, n (%)	3 (7.0)	19 (4.4)	0.434
Sarcopenia, n (%)	13 (30.2)	28 (6.4)	<0.001
**Comorbidities**			
Hypertension, n (%)	30 (69.8)	373 (85.6)	0.007
Dyslipidemia, n (%)	25 (58.1)	384 (88.1)	<0.001
Coronary heart disease, n (%)	5 (11.6)	69 (15.8)	0.467
Stroke, n (%)	2 (4.7)	38 (8.7)	0.358
**Life habits**			
Regular walking, n (%)	8 (19.0)	110 (25.3)	0.367
Current or ex-smoking, n (%)	23 (53.5)	231 (53.0)	0.949
Current or ex-drinking, n (%)	12 (27.9)	131 (30.0)	0.770
**Blood measurements**			
Albumin, g/dL	3.8 [3.5, 4.0]	4.3 [4.1, 4.5]	0.004
AST, U/L	21 [17, 27]	21 [17, 28]	0.648
ALT, U/L	14 [10, 22]	19 [14, 30]	0.001
γGP, U/L			
Fasting plasma Glucose, mg/dL	132 [110, 153]	131 [118, 154]	0.573
Glycated hemoglobin, %	6.9 [6.5, 7.6]	6.9 [6.4, 7.4]	0.655
LDLcholesterol, mg/dL	95 [79, 105]	102 [82, 120]	0.025
HDL cholesterol, mg/dL	54 [44, 70]	53 [46, 62]	0.358
Triglycerides, mg/dL	73 [54, 106]	107 [76, 156]	<0.001
Creatinine, mg/dl	0.8 [0.65, 1.01]	0.83 [0.69, 1.00]	0.728
eGFR, ml/min/1.73m^2	63.4 [43.3, 79.6]	63.2 [51.4, 75.8]	0.847
**Glocose-lowering drugs**			
Insulin, n (%)	16 (37.2)	116 (26.6)	0.138
GLP-1 receptor agonist, n (%)	2 (4.7)	32 (7.3)	0.513
Sulfonylurea, n (%)	7 (16.3)	42 (9.6)	0.170
Glinide, n (%)	12 (27.9)	106 (24.3)	0.602
Biguanide, n (%)	18 (41.9)	221 (50.7)	0.269
DPP4 inhibitor, n (%)	27 (62.8)	267 (61.2)	0.842
Pioglitazone, n (%)	8 (18.6)	139 (31.9)	0.072
α-glucosidase inhibitor, n (%)	15 (34.9)	75 (17.2)	0.005
SGLT2 inhibitor, n (%)	3 (7.0)	97 (22.2)	0.019

Data are expressed as median [25%, 75%], Mean ± SD or number (%). GNRI, geriatric nutritional risk index; CONUT, controlling nutritional status; P, provability; BMI, body mass index; SMI, skeletal mass index; AST, aspartate aminotransferase; ALT, alanine aminotransferase; LDL, low-density lipoprotein; HDL, high-density lipoprotein; eGFR: estimated glomerular filtration rate; GLP-1, lucagon-like peptide-1; SGLT2, sodium–glucose cotransporter 2.
