# Supplementary figures and images for: A nutritional assessment tool, GNRI, predicts sarcopenia and its components in type 2 diabetes mellitus: A Japanese cross-sectional study
Source: Front Nutr. 2023 Feb 1;10:1087471. doi: 10.3389/fnut.2023.1087471 (PMC9928854; doi:10.3389/fnut.2023.1087471)

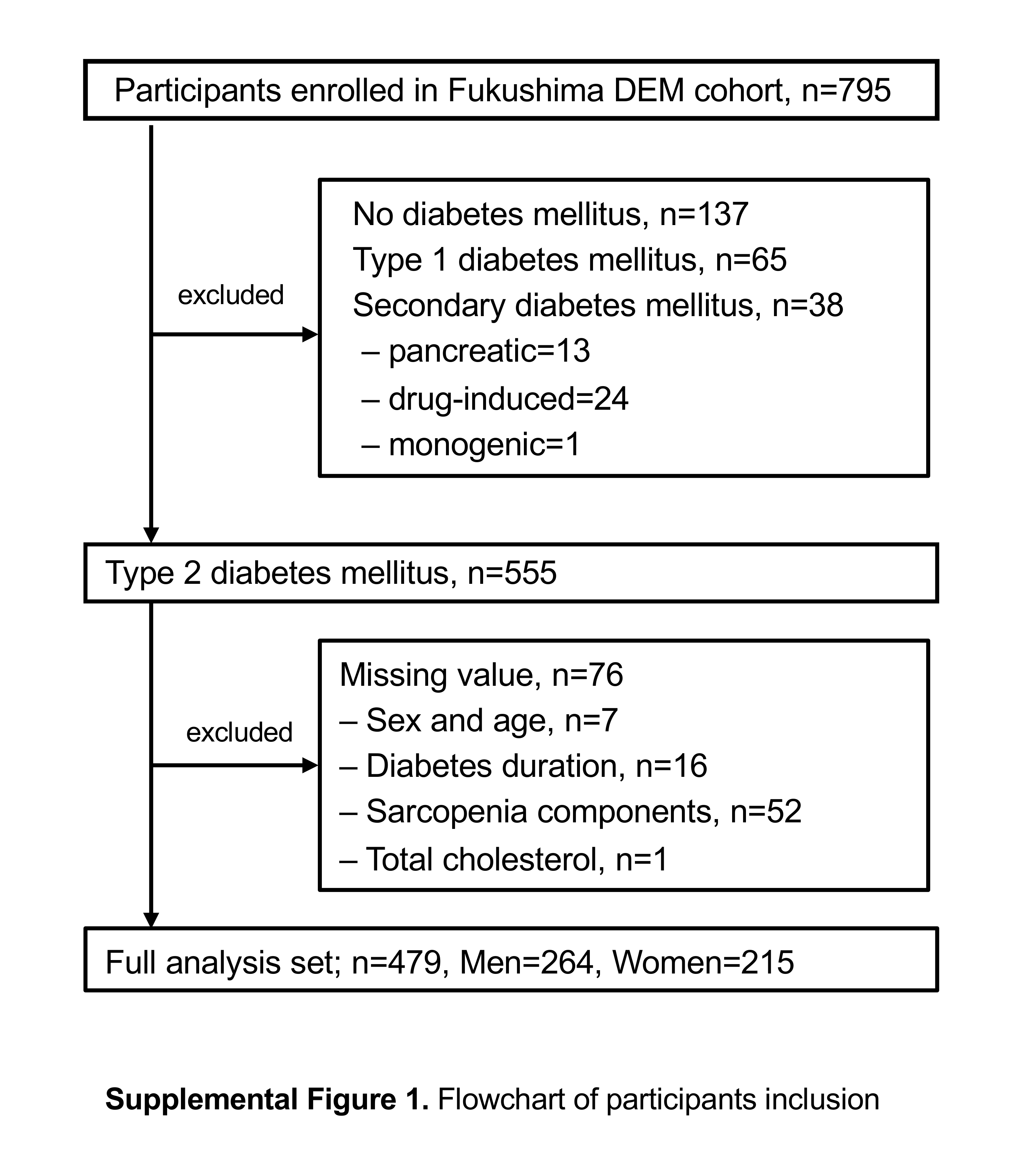

Supplement: Supplementary file 4 [file Image_1.tif]
